# Developmental trajectories of conduct problems and time-varying peer problems: the Bergen child study

**DOI:** 10.1007/s00127-024-02644-y

**Published:** 2024-03-01

**Authors:** Lisa-Christine Girard, Tormod Bøe, Sondre Aasen Nilsen, Kristin Gärtner Askeland, Mari Hysing

**Affiliations:** 1 Department of Special Needs Education, University of Oslo, Sem Saerlands vei Helga Engshus, Oslo, Norway; 2 Department of Psychosocial Science, University of Bergen, Bergen, Norway; 3 Regional Centre for Child and Youth Mental Health and Child Welfare, NORCE Norwegian Research Centre, Bergen, Norway

**Keywords:** Conduct problems, Peer problems, Childhood, Adolescence, Developmental trajectories, Bergen child study, youth@hordaland

## Abstract

**Background:**

While it is increasingly acknowledged that conduct problems and peer problems often co-occur in development, less is known about the ways in which peer problems may alter the developmental course of conduct problems for distinct subgroups.

**Methods:**

Using data from a large population-based study in Norway (the Bergen Child Study/youth@hordaland; 47.4% males), we estimated group-based trajectories of conduct problems and the presence of time-varying peer problems on the developmental progression of conduct problems between seven and 19 years of age. Risk factors for group membership were also examined.

**Results:**

A 3-group model of conduct problems best fit the data (non-engagers, low-engagers, moderate-stable). The presence of peer problems increased the estimated level of conduct problems for both the low-engagers and moderate-stable groups across adolescence. No differences in conduct problems were observed when peer problems were present in childhood or preadolescence for these two groups, nor for the non-engagers group at any point. Being male, having lower perceived economic wellbeing, and lower levels of parental education predicted group membership for the moderate-stable group, whilst lower paternal education predicted membership for the low-engagers group.

**Conclusions:**

Support for developmental ‘turning points’ was found, suggesting that adolescence is a particularly salient time for those with conduct problems. In particular, the presence of peer problems can increase observed conduct problems at this stage in development.

**Supplementary Information:**

The online version contains supplementary material available at 10.1007/s00127-024-02644-y.

## Introduction

Conduct problems, characterised by aggressive and non-aggressive behaviours that violate social norms and values, are of a growing public health concern given their high contribution towards the global mental health burden in children and adolescents [1]. At the same time, the number of children experiencing longitudinal peer problems is considered substantial, with recent reports of children on ‘emerging/increasing’ and ‘high-chronic’ trajectories between 17–25% and 4–24%, respectively [2–4]. Peer problems are characterised by difficulties within social relationships with others of a similar developmental age in which the individual may be victimised by others or become socially isolated. The plethora of associated negative life outcomes for both conduct problems and peer problems crosses multiple domains of living in adolescence and adulthood, including social, health, socioeconomic, and criminal [5, 6].

The co-occurrence of conduct problems and peer problems in studies with children and adolescents has been observed in some, but not all, groups (for a review, see [7]). This has resulted in greater attention towards (1) unpacking the direction of association (i.e., whether peer problems are an antecedent or outcome of conduct problems), and (2) trying to better understand sensitive periods when co-occurrence is likely to emerge. Results of this first line of inquiry have supported bi-directional associations, with effect sizes of similar magnitude in both directions [e.g.,8]. Results of the second line suggest that (1) co-occurrence may already present by 4 years of age for subgroups of children exhibiting moderate to high levels of both conduct problems and peer problems, and (2) while mid-childhood may be a point of decreasing trajectories for some subgroups with moderate early levels of conduct problems, peer problems in these same children appear to remain stable and/or increase into adolescence [9–11]. However, there remains a dearth of research to date that has specifically focused on the question of ‘turning points’, particularly when examining trajectories of conduct problems. Turning points in development are transitional periods or events that can alter behaviour, affect, cognition, or context, and result in lasting changes in psychological functioning and life trajectories [12, 13]. In a developmental psychopathology framework, the transition into adolescence is understood as a period of considerable change for both the individual and their surroundings, with associated turning points that have the potential to alter the developmental course [14]. As turning points can be especially salient in such transitional periods [12], investigating possible turning points in the trajectories of conduct problems in the transition from childhood to adolescence can shed light on important developmental processes. In the current context, the question is whether the presence of peer problems at differing stages of development would result in any changes within the developmental course of conduct problems. This question warrants particular attention, given the ensuing implications for preventative programming, and the compounding effects of consequent co-occurring problems for long-term maladaptive outcomes.

### Scandinavian context

Whilst the estimated prevalence of disruptive behavior disorders (including both conduct disorder and oppositional defiant disorders) have an estimated prevalence of 5.7% worldwide [15], reported rates are generally lower in Scandinavian contexts. For example, estimated prevalence for disruptive behavior disorder in Norway is reported as slightly above 2% in childhood [16, 17], and slightly above 1% in adolescence [18].

The reason for lower rates of disruptive behaviour disorders in the Scandinavian countries is not certain. Possible explanations for differences in conduct problems may stem from differences in instrumentation and reporting thresholds [e.g [19–21]]. However, one study comparing conduct problems in Norway and the UK suggested a genuine difference in rates, not indicative of reporting thresholds and/or bias [17]. Alternate explanations may relate to the social and economic situation in Norway, with the relatively low poverty and unemployment rates, small income inequalities, and higher education levels [22, 23]; coupled with ranking 2nd on overall child well-being compared to other developed countries [24]. Regarding peer problems, there is also a low prevalence across Scandinavian countries compared to other European countries [25]. With some notable exceptions, few studies using Norwegian cohorts have modelled trajectories of conduct problems, capturing the transition between childhood and adolescence [26–28], and none have examined the association with time-varying peer problems for trajectory progression. Thus, the questions of heterogeneity in the developmental progression of conduct problems across subgroups, and potential turning points arising from the presence of peer problems, remain open.

### Antecedent factors

Generally, conduct problems are higher among boys than girls [29]. However, more nuanced sex differences in trajectories have been reported using Norwegian cohorts [26, 30]. Despite being a wealthy country where income inequality is low and absolute deprivation is rare [22, 31], socioeconomic gradients (income and education) in health, including childhood conduct and peer problems, are well established in Norway, and documented within the Bergen Child Study [32]. Lower socioeconomic status has also been related to high levels of family stress and poorer parenting practices [33, 34], which could contribute to the onset and maintenance of conduct problems [35]. In this context, we examined sex, perceived family economy, and parentals levels of education as antecedents of trajectory group membership.

### Aims

We focus on three aims. First, to examine developmental trajectories of conduct problems from mid-childhood to late adolescence using a Norwegian cohort. Second, to identify whether included risk factors differentially distinguish between subgroups identified. Finally, to examine whether the presence of peer problems at specific times in development are associated with changes in the developmental course of conduct problems within the estimated trajectory groups.

## Methods

Participants in this study included 3,675 children and their parents initially enrolled in the Bergen Child Study (BCS), and the youth@hordaland study at wave 4. The BCS included 3 waves of data collection starting in 2002, which aimed to better understand the mental health and wellbeing of children born between 1993 and 1995 in Bergen, Norway. All children in private and public schools in the county of Bergen were invited for the BCS during the three first waves. The fourth wave included invitations to adolescents from a broader geographical area (Hordaland). Data collection occurred approximately every three years, when children were ages seven-nine (wave 1), 11–13 (wave 2), 14–16 (wave 3), and then again when youth were 17–19 (wave 4, youth@hordaland). Sampling selection for both the BCS and youth@hordaland have been extensively described elsewhere [e.g., [16, 36]].

Inclusion criteria in the current study included participants whose parent/(s) also participated given the use of parent-reported conduct problems as the main outcome of interest (wave 1, *n* = 7,007; wave 2, *n* = 5,173; wave 3, *n* = 1,516; wave 4, *n* = 1,790), and having at least two behavioural data points (*n* = 3,675), to reduce the possibility of over-estimation of elevated trajectory groups (i.e., those with the highest levels of conduct problems are more likely to have missing data across time). A sensitivity analysis was however also conducted with all participants who had at least 1 assessment of behaviour (*n* = 4,691), supporting the main model findings (see supplementary material). A total of 827 and 170 participants had complete data across three and four waves respectively. Demographic characteristics and descriptive statistics for the included participants can be found in Table 1. Each of the study waves were approved by the Regional Committee for Medical and Health Research Ethics in Western Norway. For the first three waves, written informed consent was obtained from all parents whose child was included in the present study. For the fourth wave, parents and adolescents provided informed consent for their own participation, and adolescents further consented to having their parents participate.

**Table 1:** Demographic and descriptive characteristics of the sample

	Wave 1	Wave 2	Wave 3	Wave 4
Male	1,740 (47.4%)			
Perceived family SES (poor/very poor)		75 (2.0%)		
Maternal education (primary or secondary only)		1,544 (42.0%)		
Paternal education (primary or secondary only)		1,662 (45.2%)		
Peer problems	0.97 (1.54)	1.03 (1.62)	1.41 (1.82)	1.41 (1.70)
Conduct problems	0.82 (1.16)	0.74 (1.07)	0.87 (1.16)	0.83 (1.12)

*Note* Frequencies and respective percentages (%) are presented for participant characteristics of the included sample, *n* = 3,675. Means and standard deviations (SD) for conduct problems and peer problems are presented. Scores on these subscales range between 0 and 10

### Outcome and time-varying covariate

Trajectories of conduct problems were assessed using the parent version of the Strengths and Difficulties Questionnaire (SDQ; [37]) across all four waves. The SDQ is a behavioural screening tool for mental health difficulties which includes the following five subscales: conduct problems, hyperactivity/inattention, emotional symptoms, peer problems, and prosocial behaviours. Five items are included for each subscale, rated on a 3-point scale from 0 (*not true*) to 2 (*certainly true*). Total possible subscale scores range from 0 to 10. The parent reported SDQ has been extensively validated within the literature and using the BCS [e.g [38, 39]], and recently studies have also provided support for the parent reported SDQ when used with older adolescents [40, 41]. Whilst the full range of the conduct problems subscale was used in the trajectory estimation (i.e., 0–10), we used previously suggested cut-offs normed in Denmark from over 63,000 parent reports to descriptively interpret trajectories as follows: low conduct problems (0–2), moderate conduct problems (3), and high conduct problems (4–10) [42]. We also used the peer problems subscale of the SDQ as a time-varying covariate within the model. Example items on this subscale include ‘*picked on or bullied by other children*’ and ‘*rather solitary, tends to play alone*’. Scores on the peer problems scale were dichotomised as low (0–2), and high (3–10), using the same suggested cutoffs. Ordinal alphas at waves 1, 2, 3 and 4 for conduct problems in the current study were 0.78, 0.82, 0.82 and 0.74, respectively, and for peer problems were 0.76, 0.79, 0.75, and 0.70, respectively.

### Time-stable covariates

To better understand characteristics associated with the probability of group membership within the estimated trajectories, we included the following time-stable covariates at wave 1: the child’s sex (boy, girl) as identified through the participant’s personal identity number in the Norwegian National Register, and at wave 2: perception of economic wellbeing as rated by the parents (very poor, poor, average, good, very good), highest level of maternal education (primary or secondary level only, which included elementary or high school education, college or university), and highest level of paternal education (primary or secondary level only, which included elementary or high school education, college or university).

### Statistical analysis

We estimated trajectories of conduct problems with both time stable and time varying covariates using the group-based trajectory model (GBTM) approach [43]. GBTM uses finite mixture modelling to model the evolution of a developmental phenomenon, in our case conduct problems. An advantage of this semi-parametric person-centered approach is the ability to identify heterogeneity between clusters of children with distinct developmental progressions of conduct problems over time, including low-frequency trajectory groups. The inclusion of time stable covariates provides greater understanding around factors, preceding the developmental trajectory, that distinguish between group membership. On the other hand, the inclusion of our time-varying covariate (i.e., peer problems) allows us to model trajectories of conduct problems and test whether the occurrence of peer problems at a particular point in time modifies or alters the developmental course of conduct problems. More specifically, the question of whether there are ‘turning points’ for children following particular trajectories of conduct problems is addressed. Because the estimates are specific to the individual trajectory groups, we are then able capture any within group differences. An important distinction is that with this approach, we are not modelling whether the occurrence of peer problems results in individual changes to group membership, but rather whether peer problems results in a change in the developmental course of conduct problems within the identified groups [43].

Estimation of model parameters uses maximum likelihood, with a quasi-newton procedure for maximization [44]. Inspection of the data revealed an extreme right skew for conduct problems, which is consistent with the use of Poisson models [43]. However, given the further high 0 values within the data, we used a zero-inflated Poisson model. The Bayesian Information Criteria (BIC) and the Akaike Information Criterion (AIC) were used to assess the best model fit in the first stage of model selection. A larger more positive value of the BIC (i.e., closer to zero) indicates a better model fit (see [43, 45]). Good model fit is further indicated by the average posterior probabilities of group membership by trajectory membership group (APP), and the odds of correct classification (OCC). These indices are calculated post model estimation, and relay information about potential group classification error. The APP specifies the likelihood that a participant with a specific behavioural profile belongs to the model’s *j* trajectory group, with an upper bound of 1. The suggested group assignment threshold for the APP is greater than 70% [43]. The OCC represents the odds that a participant has been properly classified into trajectory group *j*, better than by chance alone, with an assignment threshold suggested as greater then 5.

We used a multi-stage modelling approach to identify the best model fit for the data. First, we ran two-through five-group models comparing the BIC and AIC of each model with quadratic growth (Table 2). Given the small improvement in the BIC and AIC between the four- and five-group models, and in line with the principal parsimony, we did not estimate larger group models. The BIC and AIC suggested the best model fit for the data was the three-group model. The next step of fitting polynomial terms (constant, linear, and quadratic growth) with both time stable and time varying covariates revealed the three-group model with constant and quadratic growth only, provided the best fit for the data. Inspection of the APP and OCC further supported the three-group model (Table 3), suggesting an arguably low potential of classification error within the selected model. All analyses were conducted using Stata v17.0, using the proc traj plugin. The group-based trajectory models were visualized using the R-package *ggplot2* [46]. The term significance is used in lieu of statistical significance hereafter.

**Table 2:** Assessment of model fit: a comparison of the Bayesian information criteria (BIC) and the Akaike information criterion (AIC)

	BIC_Assessments_	BIC_Sample_	AIC
2-group	-9702.19 (*N* = 8344)	-9696.86 (*N* = 3675)	-9656.50
3-group	**-9666.41** (*N* = 8344)	**-9657.39** (*N* = 3675)	**-9589.09**
4-group	-9696.43 (*N* = 8344)	-9683.72 (*N* = 3675)	-9587.47
5-group	-9732.57 (*N* = 8344)	-9716.17 (*N* = 3675)	-9591.98

*Note* BIC_sample_ represents the actual sample size of participants included in the trajectories, the larger BIC_assesssments_ represents the total number of assessments used within the estimation of the model across time and participants. The two presented BIC scores bracket the theoretically correct BIC score (Nagin, 2005)

**Table 3:** Post model fit estimation: Trajectories of conduct problems

Trajectory Group	Average Posterior Probability of Group Membership	Odds of Correct Classification
1	71.5	33.9
2	84.3	42.2
3	78.5	311.6

## Results

### Group-based trajectories of conduct problems

A three-group trajectory model was identified. Group 1, labelled as ‘non-engagers’, comprised an estimated 30.1% of the sample. This group was characterised as having no reported conduct problems across any wave. Group 2 was comprised of an estimated 60.6% of the sample and were labelled as ‘low-engagers’. Group 2 followed a quadratic trajectory despite the level of conduct problems remaining low across time. More specifically, there was a slight reduction in reported conduct problems between childhood and preadolescence, which then rose back to initial childhood levels in adolescence and late adolescence. Group 3 comprised an estimated 9.3% of the sample and were labelled ‘moderate-stable’. Similar to Group 1, conduct problems in this group remained stable across time, albeit at higher levels (i.e., moderate as compared to none). See Table 4 for trajectory parameter estimates.

**Table 4:** Trajectory parameter estimates: 3-group conduct problems with time-varying peer problems

Group	Parameter	Estimate	SE	OR [95% CI]	*T*	*p*
1	Intercept	-2.62	0.35		-7.51	<0.001
	Peer problems	0.71	0.37	2.03 [0.99–4.17]	1.94	0.052
2	Intercept	0.54	0.19		2.86	0.004
	Linear	-0.15	0.04		-4.11	< 0.001
	Quadratic	0.01	0.00		4.04	< 0.001
	Peer problems	0.63	0.05	1.88 [1.71–2.06]	13.43	< 0.001
3	Intercept	0.81	0.07		11.09	< 0.001
	Peer problems	0.48	0.07	1.62 [1.43–1.84]	7.42	< 0.001

*Note N* = 3675. The non-engagers are Group 1, the low engagers are Group 2, and the moderate stable are Group 3

### Time stable and time varying covariates

**Fig. 1:**
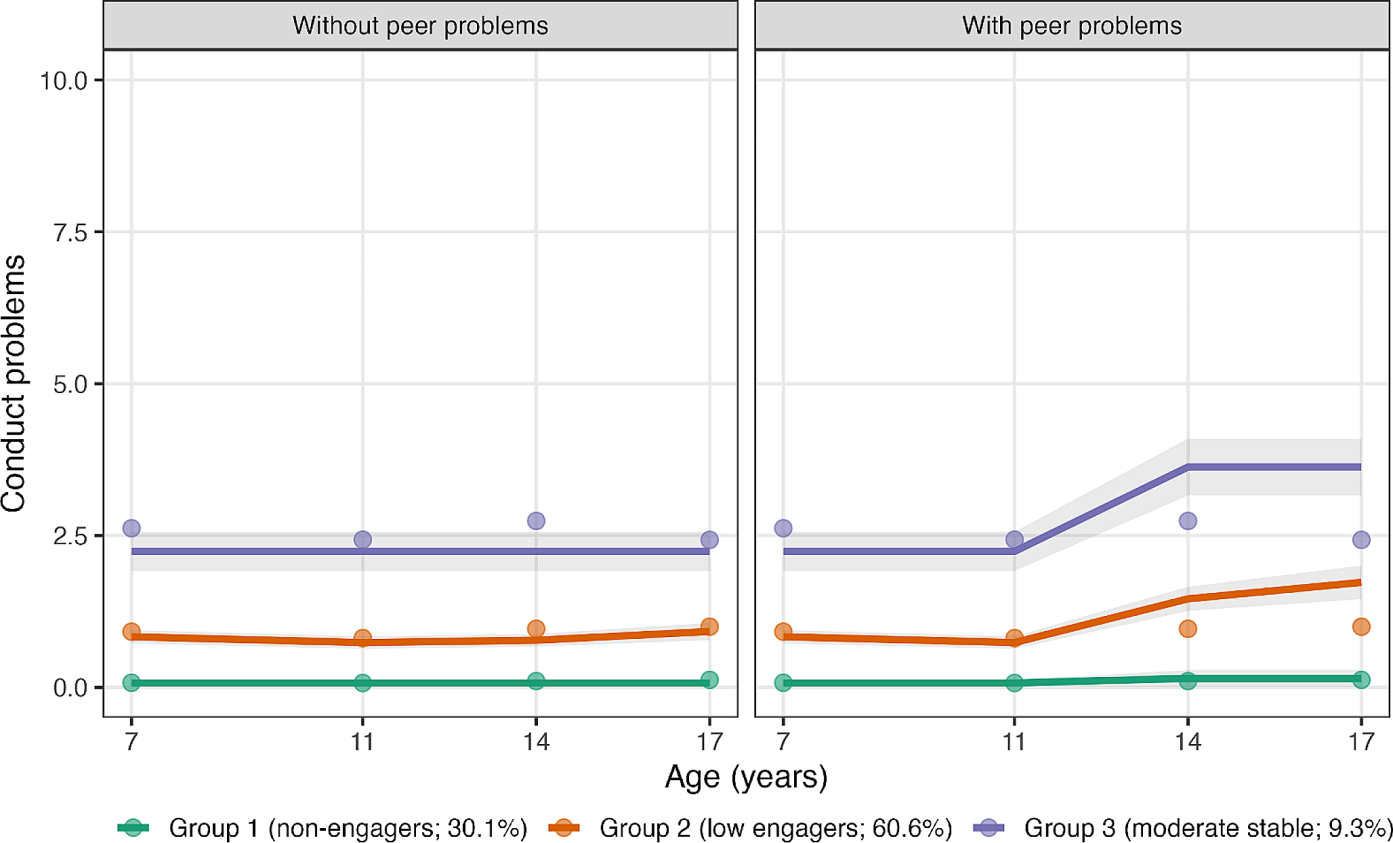
*Note*Trajectories of conduct problems from childhood to late adolescence: with and without time covarying peer problems. Trajectories of conduct problems without (left panel) and with (right panel) time covarying peer problems. The shaded grey areas represent the 95% pointwise confidence intervals of the estimated trajectories. The points represent the observed means of conduct problems across groups

Examination of time stable factors that distinguished between group membership included sex, perceived family economic wellbeing, along with the highest level of maternal and paternal education. Group 1, the non-engagers were used as the comparison group. The only factor that was significant in predicting group membership for the ‘low-engagers’ group was paternal education. That is, participants with fathers who had primary or secondary level education only had a higher likelihood of membership in Group 2. In contrast, sex, perceived family economic wellbeing, along with maternal and paternal level of education were all significant predictors of group membership in the ‘moderate-stable’ group. More specifically, being male, having ‘poor or very poor’ perceived family economic wellbeing, and having mothers and fathers with primary or secondary level education only, increased the risk of membership in Group 3 (Table 5). Regarding the time varying factor, the presence of peer problems in adolescence resulted in a statistically significant change to the developmental trajectory of conduct problems, in particular for Groups 2 and 3 (See Fig. 1 for trajectories of conduct problems with and without peer problems, along with Table 4 for parameter estimates). For these groups, the presence of peer problems increased the estimated level of conduct problems in adolescence and late adolescence within the estimated trajectories.

**Table 5:** Time stable risk factors by group membership

	Estimate	SE	*T*	*P*
Sex (male)				
Group 2	0.14	0.11	1.25	0.213
Group 3	0.63	0.19	3.29	0.001
Economic wellbeing (poor/very poor)				
Group 2	1.85	1.39	1.33	0.183
Group 3	3.03	1.26	2.40	0.017
Maternal Education (primary/secondary only)				
Group 2	0.03	0.13	0.21	0.836
Group 3	0.47	0.20	2.32	0.020
Paternal Education (primary/secondary only)				
Group 2	0.30	0.13	2.27	0.023
Group 3	0.77	0.20	3.89	0.001

*Note* The non-engagers (Group 1) are the reference category

## Discussion

Developmental trajectories of conduct problems across childhood and adolescence have been well studied, however minimal attention has been paid to the ways in which the presence of peer problems may be associated with changes in the course of its progression for distinct subgroups. Moreover, there is a scarcity of studies examining heterogeneity in the longitudinal progression between subgroups of children with conduct problems, including antecedent risk factors for group membership, within a Norwegian context. Our results contribute important new insights in these areas and are discussed in turn.

### Developmental trajectories of conduct problems

First, using a large longitudinal population-based study in Norway, we found support for a 3-group model of conduct problems as best fitting the data, with just over 90% of the cohort following trajectories described by non-existent or low levels of conduct problems. This is a much higher percentage as compared to previous studies outside Norway, which find around 60% of sampled populations to be following persistently low levels of conduct problems across childhood and adolescence [47–50]; although consistent with one study’s findings within Norway where close to 85% of the sample were following a persistently low trajectory [28]. Further, in contrast to previous studies we found no evidence of a chronically high engaging group. Rather, we only found support for a group (less than 10% of the sample) following a trajectory of moderate stable levels of conduct problems across time, which is similar to the 3-group model of aggression found in a Canadian national cohort [51]. Our results provide little support for Moffitt’s [52] developmental dual taxonomy (i.e., life-course persistent and adolescent onset) within this Norwegian population-based sample. While we did find that the moderate-stable group was persistent in their engagement of conduct problems across mid-childhood to late adolescence, potentially consistent with a life-course persistent group, the level of conduct problems remained at the low end of moderate rather than high. Moreover, no support for an adolescent onset group was found when examining trajectories of conduct problems alone. This is perhaps not surprising in the context of the recent findings by Bøe et al., [18] demonstrating lower estimated prevalence of conduct problems in the adolescent period, as compared to childhood in a Norwegian cohort [16, 17]. Taken together, our results build upon this previous work by examining the estimated prevalence and heterogeneity in the presentation of trajectories of conduct problems across subgroups, from seven to 19 years, demonstrating support for what was previously labelled the ‘Nordic advantage’ [17] in externalising difficulties such as conduct problems.

### Antecedent risk factors for group membership

To better understand factors that may distinguish between group membership in identified trajectories of conduct problems, we examined factors that have been previously implicated in children and adolescents’ mental health difficulties within the Norwegian context. Male sex was a predictor of the moderate-stable group, which is in line with some previous findings that conduct problems are more prevalent among boys in the Norwegian context [28, 30]. Low parental education levels and poorer economic well-being were predictors of the moderate-stable group. In line with this finding, Gutman et al. [49] found socioeconomic risk factors to be related to problem pathways that were both higher and more lasting. Accumulation of several risk factors, such as comorbid mental health problems in addition to socioeconomic adversity, has also been related to more persistent conduct problem trajectories [49]. This agrees with the current findings of more risk factors in the moderately stable group relative to the low engagers group.

### The presence of peer problems: adolescence as a turning point

Given the wealth of studies evidencing the co-occurrence of conduct problems and peer problems across development [e.g., [7–11]], we examined the inclusion of time varying peer problems within the estimated trajectories of conduct problems to determine whether the presence of peer problems would be associated with changes in the developmental course of conduct problems for subgroups identified, and if so, at which points in development. To the best of our knowledge, this is the first study to specifically address this question. Our results suggest that for Group 1, the non-engagers, the presence of peer problems was not a significant factor associated with changes in the estimated trajectory of conduct problems. Thus, the presence of elevated peer problems for those with non-existent levels of conduct problems did not result in any changes in conduct problems across time. For Groups 2 and 3, a significant deviation in the developmental trajectory of conduct problems was found when peer problems were present. Closer visual examination of these two trajectory groups revealed minimal associated change to the estimated trajectories of conduct problems for both groups during childhood and preadolescence. However, for both groups, the presence of elevated peer problems was associated with a modified estimated developmental course of conduct problems between preadolescence and adolescence, subsequently increasing the levels of conduct problems, which continued to either increase (Group 2) or remain stable (Group 3) thereafter, between early and late adolescence. This would suggest that adolescence may be a particularly salient time in development whereby the presence of elevated peer problems may have a more detrimental associated influence on engagement in conduct problems for those who initially display with conduct problems, even at low and moderate levels. These results are theoretically well-aligned with an ‘adolescent-onset’ group within the dual taxonomy [52], given that the mechanisms suggested are centrally related to the peer group and context. Indeed, the influence of peers on choices and behaviours grows stronger during adolescence. The importance of peers in escalating conduct problems may be related to a range of different processes, including negative interaction patterns in which conduct problems may be a response to negative peer actions such as bullying, and may be part of a negative self-perpetuating cycle. Exclusion from peers may also lead to deviant peer affiliations, which again may further increase the risk of conduct problems.

### Limitations

Several limitations are addressed for interpretating the study findings. First, attrition across waves resulted in a reduced sample used in the current study. Whilst sensitivity analyses were conducted and supported the main model findings (see supplementary material), the sample used may still be less representative as compared to the originally recruited sample. The exact nature of the attrition across all four waves is uncertain and complicated by the fact that the fourth wave had a different target population than the earlier waves. Still, studies on waves 1 and 2 have identified low parental education, non-traditional family forms, and child psychological problems to be associated with parental non-participation [53, 54], which should be kept in mind when interpreting the results of this study. Second, implications of shared method variance must be considered given the sole reliance on parent report for both assessment of child/adolescent conduct problems and peer problems, along with antecedent risk factors included within the model. Ideally, the use of multiple respondents would have strengthened the robustness of findings. Third and relatedly is the use of parent reports for the SDQ at wave 4 given the age of participants (i.e., 17–19 years). Whilst the SDQ has been normed for adolescents up to the age of 16, the use of the SDQ has been validated, including for use with older adolescents, lending support for its current use [40, 41, 55]. Nonetheless, parents may still be less reliable informants with respect to the adolescent’s mental health, and in particular experiences of peer problems, at this age as compared to the youth themselves. Finally, most of the time stable risk factors were collected at wave 2 rather than wave (1) Theoretically, the inclusion of time stable risk factors should precede the first time point of the trajectory in question. Arguably, our time stable risk factors of interest should however be relatively stable across waves 1 and 2. Replication of findings which address these noteworthy limitations is nevertheless warranted.

## Conclusions

Taken together, results of the current study shed important new insights of practical value. We identified adolescence as a developmental period in which the presence of peer problems may result in important changes for already present conduct problems, even for those following trajectories at low levels. This may suggest that school programming efforts focused on building social skills and positive peer relationships may be especially effective in mid-childhood and preadolescence, just prior to the adolescent period. Our results also support previous findings related to risk factors for elevated groups (i.e., being male, lower economic wellbeing, lower maternal education), whilst further highlighting paternal education levels lend to increased risk of membership in elevated groups and ought not to be ignored within the literature. Future directions include building upon the current work by examining whether the presence of peer problems at differing stages of development would result in similarly identified turning points within developmental trajectories of conduct problems for boys and girls separately.

## Electronic supplementary material

Below is the link to the electronic supplementary material.

**Supplementary Material 1**: Developmental trajectories of conduct problems and time-varying peer problems: the Bergen child study

## Data Availability

There are legal and ethical restrictions on sharing the dataset used for the present manuscript. Access to the dataset require application and approval by the Regional Committee for Medical and Health Research Ethics (REC) in Western Norway and the Norwegian Centre for Research Data (NSD). In addition, the Norwegian Health research legislation and the Norwegian Ethics committees require explicit consent from participants in order to transfer health research data outside of Norway. Data are from the Bergen Child Study and the Norwegian youth@hordaland study, owned by NORCE Norwegian Research Centre. For more information, please contact bib@norceresearch.no.
