# Supplementary material for: Developmental trajectories of conduct problems and time-varying peer problems: the Bergen child study
Source: Soc Psychiatry Psychiatr Epidemiol. 2024 Mar 1;59(12):2237–46. doi: 10.1007/s00127-024-02644-y (PMC11522142; doi:10.1007/s00127-024-02644-y)
Supplement: Supplementary file 1 — Supplementary Material 1: Developmental trajectories of conduct problems and time-varying peer problems: the Bergen child study [file 127_2024_2644_MOESM1_ESM.docx]

**Supplementary Material**

TableS1. Post Model Fit Estimation: Trajectories of Conduct Problems

| Trajectory Group | Average Posterior Probability of Group Membership | Odds of Correct Classification |
| --- | --- | --- |
| 1 | 69.0 | 24.7 |
| 2 | 81.3 | 24.6 |
| 3 | 77.1 | 236.7 |

Note: *N* = 4691. Average Posterior Probability of Group Membership greater than 70 and an OCC greater than 5 represents a good model fit.

TableS2. Trajectory Parameter Estimates: 3-Group Conduct Problems with Time-Varying Peer Problems

| Group | Parameter | Estimate | SE | *T* | *p* |
| --- | --- | --- | --- | --- | --- |
|  |  |  |  |  |  |
| 1 | Intercept  Peer problems | -2.56  0.74 | 0.33  0.33 | -7.65  2.24 | <0.001  0.025 |
|  |  |  |  |  |  |
| 2 | Intercept | 0.38 | 0.18 | 2.08 | 0.038 |
|  | Linear  Quadratic  Peer problems | -0.12  0.01  0.64 | 0.03  0.00  0.04 | -3.32  3.28  14.86 | 0.001  0.001  <0.001 |
|  |  |  |  |  |  |
| 3 | Intercept | 0.83 | 0.07 | 11.66 | <0.001 |
|  | Peer problems | 0.49 | 0.06 | 7.87 | <0.001 |

Note: *N* = 4691. The non-engagers are Group 1, the low engagers are Group 2, and the moderate stable are Group 3.

TableS2: Time Stable Risk Factors by Group Membership

|  | **Estimate** | **SE** | ***T*** | ***p*** |
| --- | --- | --- | --- | --- |
| Sex (male) |  |  |  |  |
| Group 2 | 0.16 | 0.11 | 1.46 | 0.145 |
| Group 3 | 0.74 | 0.18 | 4.06 | <0.001 |
| Economic wellbeing  (poor/very poor) |  |  |  |  |
| Group 2 | 1.42 | 0.94 | 1.51 | 0.131 |
| Group 3 | 2.49 | 0.83 | 4.06 | <0.001 |
| Maternal Education (primary/secondary only) |  |  |  |  |
| Group 2 | 0.05 | 0.13 | 0.39 | 0.699 |
| Group 3 | 0.50 | 0.19 | 2.66 | 0.008 |
| Paternal Education (primary/secondary only) |  |  |  |  |
| Group 2 | 0.32 | 0.13 | 2.60 | 0.009 |
| Group 3 | 0.81 | 0.19 | 4.31 | <0.001 |

Note: *N* = 4691. The non-engagers (Group 1) are the reference category. The low engagers are Group 2, and the moderate stable are Group 3.
